# Changes of Radiation Treatment Concept Based on ^68^Ga-PSMA-11-PET/CT in Early PSA-Recurrences After Radical Prostatectomy

**DOI:** 10.3389/fonc.2021.665304

**Published:** 2021-06-01

**Authors:** Dirk Bottke, Jonathan Miksch, Reinhard Thamm, Thomas Krohn, Detlef Bartkowiak, Meinrad Beer, Christian Bolenz, Ambros J. Beer, Vikas Prasad, Thomas Wiegel

**Affiliations:** ^1^ Xcare Praxis für Strahlentherapie, Trier, Germany; ^2^ Department of Nuclear Medicine, University Hospital of Ulm, Ulm, Germany; ^3^ Department of Radiation Oncology and Radiotherapy, University Hospital Ulm, Ulm, Germany; ^4^ Radiologie Aachen Land, Würselen, Germany; ^5^ Department of Radiology, University Hospital of Ulm, Ulm, Germany; ^6^ Department of Urology, University Hospital of Ulm, Ulm, Germany

**Keywords:** prostate cancer, biochemical recurrence, early salvage radiotherapy, positron emission tomography (PET), PSMA

## Abstract

**Background and Purpose:**

Salvage radiotherapy (SRT) is the main potentially curative treatment option for prostate cancer patients with post-prostatectomy PSA progression. Improved diagnostics by positron emission tomography/computed tomography (PET/CT) can lead to adjustments in treatment procedures (e.g. target volume of radiotherapy, androgen deprivation therapy). We analyzed the impact of ^68^Ga-PSMA-11-PET/CT on the target volume in early biochemical recurrence (PSA up to 0.5 ng/ml).

**Patients and Methods:**

We retrospectively analyzed 76 patients with biochemical recurrence after radical prostatectomy in whom SRT was planned after ^68^Ga-PSMA-11-PET/CT. All patients had a PSA ≤0.5 ng/ml. An experienced radiation oncologist determined the radiotherapy concept, first with consideration of the PET/CT, second hypothetically based on the clinical and pathological features excluding PET/CT results.

**Results:**

Without considering the PET/CT, all 76 patients would have been assigned to RT, 60 (79%) to the bed of the prostate and seminal vesicles alone, and 16 (21%) also to the pelvic lymph nodes because of histopathologic risk factors. Uptake indicative for tumor recurrence in ^68^Ga-PSMA-11-PET/CT was found in 54% of the patients. The median pre-PET/CT PSA level was 0.245 ng/ml (range 0.07–0.5 ng/ml). The results of the PET/CT led to a change in the radiotherapeutic target volume in 21 patients (28%). There were major changes in the target volume including the additional irradiation of lymph nodes or the additional or exclusive irradiation of bone metastases in 13 patients (17%). Minor changes including the additional irradiation of original seminal vesicle (base) position resulted in eight patients (11%).

**Conclusion:**

Using ^68^Ga-PSMA-11-PET/CT for radiation planning, a change in the treatment concept was indicated in 28% of patients. With PET/CT, the actual extent of the tumor can be precisely determined even with PSA values of ≤0.5 ng/ml. Thus, the treatment concept can be improved and individualized. This may have a positive impact on progression free survival. Our results warrant further prospective studies.

## Introduction

Radical prostatectomy (RP) is considered to be a standard treatment option for patients with clinically localized prostate cancer (PCa). Nevertheless, up to 50–80% of these men develop biochemical recurrence depending on risk factors such as an advanced pathological stage, a high Gleason score or positive surgical margins (1). In case of PSA recurrence, salvage radiotherapy (SRT) is the only curative option, resulting in approximately 60% of the patients reachieving an undetectable PSA. After 5 years, 80% of these men are free from progression (2). The pre-SRT PSA level is a significant factor of progression, with more favorable results for patients with low PSA levels (0.5 ng/ml or less) (3, 4). Accordingly, European guidelines (EAU) recommend early SRT at a PSA <0.5 ng/ml (5).

At PSA levels <1 ng/ml, most imaging methods are not suitable to detect the correlate for disease progression. Therefore, up to 20% of patients with SRT to the prostate bed (with or without including original seminal vesicle) without morphological correlate will be treated locally without actual local recurrence (2).

Prostate-specific membrane antigen (PSMA) is a cell surface protein with high expression in majority of prostate cancer (6). ^68^Ga-PSMA has been used since 2012 as PSMA-ligand in recurrent prostate cancer (7–9). Especially at low PSA levels, the detection rate of ^68^Ga-PSMA-11-PET/CT is significantly higher in comparison to other imaging methods.

In a retrospective analysis of 2,533 patients with biochemical progression after RP, Afshar-Oromieh et al. found that 69% of the patients had at least one positive lesion indicating PCa recurrence. The detection rates were 43% for PSA levels ≤0.2 ng/ml, 58% for PSA >0.2 to ≤0.5 and 72% for PSA >0.5 to ≤1.0. Tumor detection was clearly associated with PSA level and higher Gleason scores (8).

Recently, we reported a detection rate of 50% in 116 patients with PSA levels up to 0.6 ng/ml and in the PSA subgroups 0–0.2, 0.21–0.3, and 0.31–0.6 ng/ml; 24, 57, and 65%, respectively (9).

Bluemel et al. analyzed the impact of ^68^Ga-PSMA-11-PET/CT in patients with PSA failure and negative F-18-choline-PET/CT. Of 125 patients, 32 patients with negative F-18-choline-PET/CT received an additional ^68^Ga-PSMA-11-PET/CT, which detected sites of recurrence in 43.8% (10).

This new possibility of precise detection of PSMA-expressing lesions can lead to changes of tumor staging and radiation planning. Data from numerous studies are available, especially on the impact of tumor stage changes on salvage radiotherapy planning (**Table 2**). However, only few data are available for patients with early biochemical recurrence (PSA <0.5 ng/ml) (11).

The goal of this retrospective, single-center analysis was to assess the impact of ^68^Ga-PSMA-11-PET/CT on the radiotherapeutic treatment concept in biochemical recurrence up to PSA 0.5 ng/ml.

## Material and Methods

### Patients

In this retrospective analysis, only patients with PSA ≤0.5 ng/ml after RP and having undergone PSMA PET/CT prior to the radiation therapy were included. Patients’ data were retrospectively analyzed from the institutional database of the Department of Nuclear Medicine and the Department of Radiation Oncology which are part of the Comprehensive Cancer Center Ulm (CCCU). Overall, 76 patients were found to fulfill the inclusion criteria. For all patients SRT was planned after ^68^Ga-PSMA-11-PET/CT.

An experienced radiation oncologist determined patient’s actual treatment concept with consideration of the PET/CT images. Retrospectively a hypothetical treatment concept (prescription and planning target volume [PTV] contouring) was planned based on the clinical and pathological parameters excluding PET/CT results: In pT2- and pT3a-tumors the PTV included the prostate bed and the basis of the former seminal vesicles. In pT3b-tumors, the bed of seminal vesicles was included, too. Inclusion of regional pelvic lymph nodes was considered in case of histopathologic risk factors (e.g. pN1, Gleason score ≥8).

All patients gave their written informed consent for a retrospective analysis of their data in an anonymized form. The study was approved by the local ethics committee of Ulm University (221/20-FSt/Sta).

### Radiopharmaceutical Preparation

The ^68^Ga-HBED-CC-PSMA complex (ABX GmbH, Radeberg, Germany) was produced as already published. For labeling the 50 mCi (1,850 MBq), iThemba LABS, South Africa ^68^Ge/^68^Ga radionuclide generator was used (12, 13).

### PET/CT Imaging Protocol and Interpretation

PET/CT images were acquired by a Biograph mCT (40)S in 3D acquisition mode 63.4 ± 11.4 min after intravenous infusion of 160.3 ± 29.4 MBq ^68^Ga-PSMA-11. Axial, sagittal and coronal slices were reconstructed afterwards. For attenuation correction and anatomical correlation, a low-dose CT was performed. Bed positions were set weight-based taking circa 2.5 min per bed position for body scan and 2 min for scanning legs. Scans were done from the mid-thighs to the vertex in 5 to 8 bed positions resulting in 15 to 20 min for each scan (170.2 ± 39.7 mAs). Intravenous contrast (80 to 120 ml Ultravist 370, Bayer Schering Pharma, Berlin, Germany) and 15 to 20 mg of furosemide were administered in 71 (93%) and 68 patients (89%) unless contraindicated. For a diagnostic CT, scans were performed 70 s past contrast injection for the venous phase.

A tracer uptake more than the immediate surrounding tissue and not related or explained due to the physiological expression was considered as pathologic. Two experienced nuclear medicine physicians with more than 10 years of experience in PET/CT analyzed the images.

## Results

The median PSA level before PET imaging was 0.245 ng/ml (range 0.07–0.5 ng/ml). Median age was 67 years (range 47–79 years). Patient characteristics are shown in **Table 1**.

**Table 1:** Patients’ characteristics.

Patient number	76
Age (years), median (range)	67 (47–79)
iPSA (ng/ml), median (range)	7.31 (1.93–35.0)
Gleason score n (%)	
Low risk (≤6) (%)	13 (17.1)
Intermediate risk (7) (%)	45 (59.2)
High risk (≥8) (%)	17 (22.4)
Unknown	1 (1.3)
Initial TNM classification n (%)	
≤pT2a (%)	4 (5.3)
pT2b (%)	4 (5.3)
≥pT2c (%)	68 (89.4)
pN0 (%)	69 (90.8)
pN1 (%)	5 (6.6)
cN0	2 (2.6)
cM0	76 (100)
PSA pre-PET/CT	0.245 (0.07–0.5)

Pathological tracer uptake as a sign of tumor recurrence in ^68^Ga-PSMA-11-PET/CT was found in 54% of the patients.

Additional information from ^68^Ga-PSMA-11-PET/CT lead to adaptation of RT planning in 28% (n = 21) of cases. In the hypothetical scenario without considering PET/CT results all the76 patients would have received RT: 60 (79%) to the bed of the prostate and seminal vesicles alone, and 16 (21%) also to the pelvic lymph nodes because of histopathologic risk factors.

**Figure 1:**
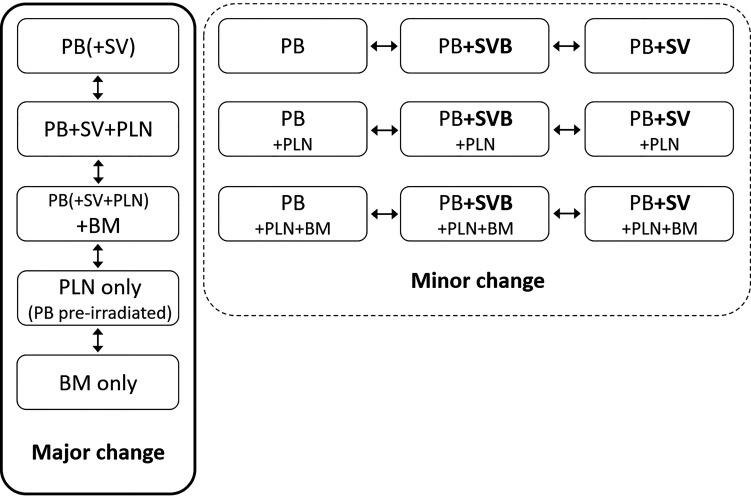
Definition of major and minor changes of radiotherapeutic treatment concept. PB, postoperative prostate bed; SV, original seminal vesicle position; SVB, original seminal vesicle base position; PLN, pelvic lymph nodes; BM, bone metastasis; NC, no change.

**Figure 2:**
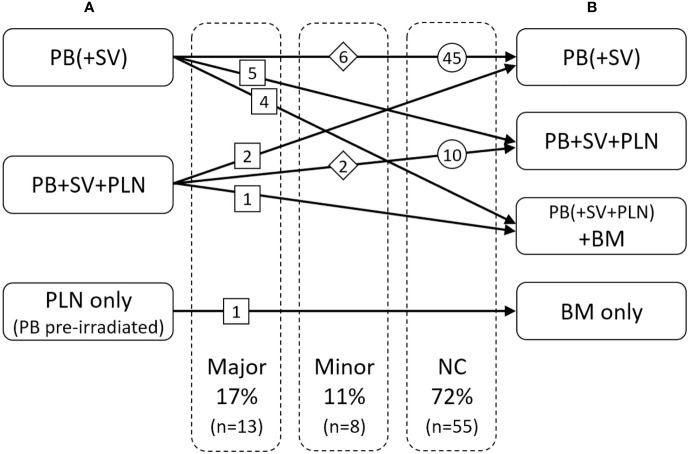
Changes of radiotherapeutic treatment concept: **(A)** based on the clinical and pathological situation without PET/CT, **(B)** with consideration of the PET/CT. PB, postoperative prostate bed; SV, original seminal vesicle position; PLN, pelvic lymph nodes; BM, bone metastasis.

**Figure 3:**
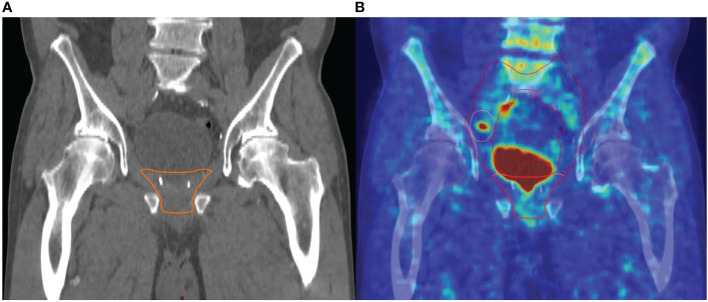
Example of major change of the radiotherapeutic treatment concept: **(A)** target volume without pelvic lymph nodes based on the clinical and pathological situation without PET/CT, **(B)** target volume with consideration of the PET/CT: additional irradiation of pelvic lymph nodes based on a PET/CT-positive right iliac lymph node metastasis.

We have defined major and minor changes. Major changes included the additional or exclusive irradiation of lymph nodes or the additional or exclusive irradiation of bone metastases based on the PET/CT. Minor changes included the additional irradiation of original seminal vesicle (base) position (**Figure 1**). Due to PET/CT, major changes were necessary in 13 patients (17%) and minor changes in eight patients (11%) (**Figures 2** and **3**). Postoperative prostate bed ± vesicle base position was irradiated with 72–74 Gy (median 72 Gy) and-positive lymph nodes with 60 Gy (range 50.4–66.6 Gy).

Based on the PET/CT, in six patients (8%), bone metastases were detected and were additionally irradiated with median 45 Gy (range 39–54 Gy).

**Figure 4:**
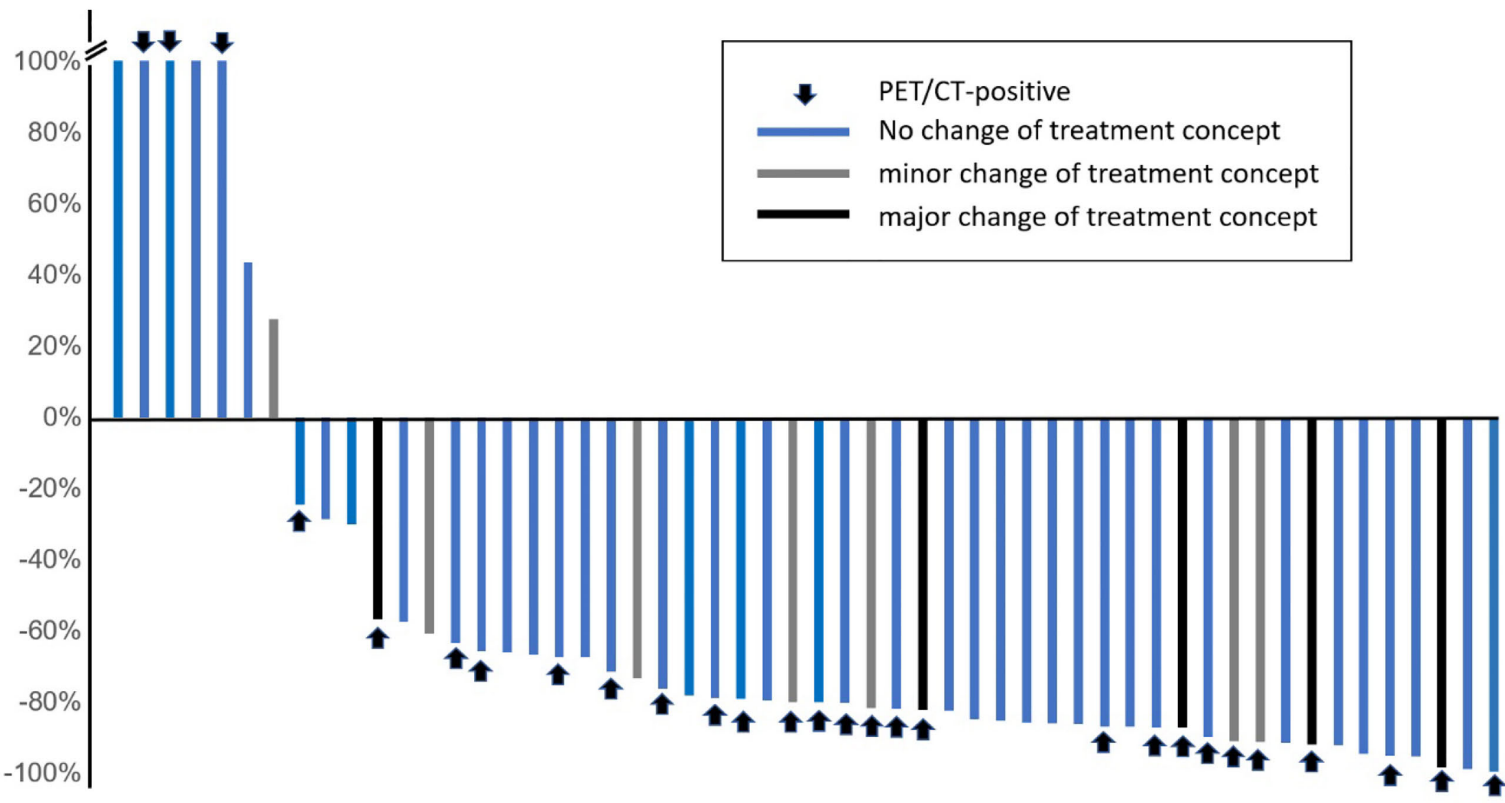
PSA changes after salvage treatment.

PSA values 6 months after completion of SRT were available in 54 patients, out of which 47 (87%) patients achieved a PSA response (**Figure 4**).

## Discussion

In our retrospective study ^68^Ga-PSMA-11-PET/CT led to changes of the RT target volume in 28% of patients with PSA ≤0.5 ng/ml after RP.

Detecting the site of prostate cancer recurrence is crucial for a successful treatment planning. In the presence of distant metastases, a prostate bed RT is not indicated. On the other hand, in the event of an exclusive loco-regional recurrence, a long-term ADT could be avoided or at least delayed by SRT. In the pre-PSMA era, most patients with PSA-failure had to undergo “blind” SRT under the assumption of local recurrence limited to the prostatic bed only.

It is equally imperative to stress that a negative PSMA-PET should not delay early SRT as the sensitivity of PSMA PET/CT for detection of micrometastases is questionable. Therefore, in the absence of any suspicious findings on PSMA-PET/CT, early SRT of the prostate bed should be offered without time delay (14).

On the other side of the spectrum, the possibility to visualize PSMA avid lesions at an early stage prior to the SRT offers the unique possibility of individualization of treatment planning as is shown in our study. ^68^Ga-PSMA-11-PET/CT led to changes of the radiation concept in 28% of all cases. All patients had a PSA <0.5ng/ml, impressively demonstrating the great potential of this imaging approach.

The impact of PSMA-PET/CT on SRT planning is extensively evaluated in several studies, albeit in heterogeneous patient population as is evident from **Table 2**. In most studies the median PSA value was significantly higher than in our study (median PSA 0.245 ng/ml).

**Table 2:** Studies assessing the impact of PSMA-PET/CT on salvage radiotherapy planning.

Authors	Year	n	Median PSA (ng/ml) (range)	PSA limit (ng/ml)	PSMA+	Extra-pelvic PSMA+	Any SRT planning change
Van Leeuwen et al. (15)	2016	70	0.2 (0.05–0.99)	<1	55%	6%	35%
Sterzing et al. (16)	2016	42	2.8 (0.16–113)	None	60%	N/A	61%
Bluemel et al. (17)	2016	45	0.67 (0.10–11.2)	None	54%	9%	42%
Albisinni et al. (18)	2017	48	2.2 (0.72–6.7)	None	N/A	N/A	76%
Habl et al. (19)	2017	83	0.69 (0.09–14.7)	None	71%	10%	57%
Koerber et al. (20)	2018	71	1.2 (0.03–41.24)	None	N/A	51%	54%
Frenzel et al. (21)	2018	75	0.2 (0.02–653.2)	None	N/A	N/A	43%
Farolfi et al. (11)	2019	119	0.32 (0.20–0.50)	<0.5	35%	21%	30%
Calais et al. (22)	2018	270	0.48 (0.03–1.0)	<1	49%	13%	19%
Schmidt-Hegemann et al. (23)	2019	62	0.44 (0.15–6.24)	None	54%	3%	50%
Boreta et al. (24)	2019	125	0.4 (0.28–0.63)	≤2.0	53%	20%	30%
Current study	2020	76	0.25 (0.07–0.50)	<0.5	54%	3%	28%

N/A, not available.

Our results are in line with the retrospective study published by Farolfi et al. (11), to our knowledge the only work that has also studied patients with low PSA limit of 0.5 ng/ml. 119 patients with a median PSA of 0.32 ng/ml (range 0.2–0.5 ng/ml) were evaluated. ^68^Ga-PSMA-11-PET/CT was positive in 41 patients (34.4%). Pathological PSMA uptake was detected in the prostate bed (three patients), in the pelvic lymph nodes (21 patients), in the retroperitoneal lymph nodes (four patients) and in bone (21 patients). The initial planned radiation concept was changed in 36 patients (30.2%) due to the PET/CT results.

In contrast, a multicenter post-hoc analysis of 270 patients with biochemical recurrence after RP with a PSA ≤1 ng/ml (median 0.48 ng/ml) showed that PSMA-PET/CT had a major impact in 19% of patients (22). A major impact was defined as PSMA-PET/CT-positive disease outside planning target volumes expanded from clinical target volumes (CTV) covering both the prostate bed and pelvic lymph nodes. The two most common PET-positive locations outside the CTV were bone (44%) and lymph nodes (31%). These results show the need for an earlier PSMA-PET/CT (22).

Habl et al. analyzed staging changes due to ^68^Ga-PSMA-11-PET and its impact on RT procedure in 100 patients after radical prostatectomy. Median PSA level was 1.0 ng/ml (range 0.12–14.7 ng/ml). 29 patients had initial pN1 disease. In 76 patients, at least one pathological PSMA uptake was found. Of these, 80% showed no morphological correlate in the corresponding CT or MRI. The tumor stage was changed in 43% of the patients. Due to the PSMA-PET/CT imaging, initial RT planning was modified in 59% of all cases. An additional simultaneous integrated boost to the prostate bed or lymph nodes was given to 32 and 63%, respectively. Ten patients received stereotactic body radiation therapy to single bone metastases (19).

Our study has some limitations due to the retrospective and monocentric approach. In addition, a biopsy of PET-positive lesions is often not feasible, particularly in patients with recurrent prostate cancer. Therefore, the lack of histological validation is a common limitation in many imaging studies.

Our results showed that ^68^Ga-PSMA-11-PET/CT is a valuable tool in the RT planning procedure of patients with recurrent PCa after radical prostatectomy even at PSA levels ≤0.5 ng/ml. They support the implementation of this imaging procedure in routine practice for biochemical progression after RP.

However, it remains unclear whether the use of PSMA-PET/CT in planning SRT could improve outcomes.

In September 2018, Calais et al. initiated a randomized phase III trial to determine whether oncological outcomes can be improved by PSMA-PET/CT in patients with early biochemical recurrence following RP. A total of 193 patients will be randomized to standard SRT (without PSMA-PET/CT) or PET scan prior to SRT planning. The primary endpoint is the biochemical progression-free survival after SRT (25).

In the future, the superior soft-tissue contrast of PET/MRI may be able to further improve the detection of pelvic tumor lesions. First results showed that the detection rate was higher than the published results for PET/CT (26). Up to now, however, the availability of PET/MRI is very low.

## Conclusion

^68^Ga-PSMA-11-PET/CT showed a high impact on radiation therapy procedure in patients with biochemically recurrent prostate cancer at PSA levels <0.5 ng/ml. With 28% changes in radiotherapy planning, ^68^Ga-PSMA-11-PET/CT is an important tool in guiding radiation treatment in this patient group. However, clinical data about the outcome of those treated patients have to be awaited.

## Data Availability Statement

The raw data supporting the conclusions of this article will be made available by the authors, without undue reservation.

## Ethics Statement

All patients gave their written informed consent for a retrospective analysis of their data in an anonymized form. The study was approved by the local ethics committee of Ulm University (221/20- FSt/Sta).

## Author Contributions

RT, TW, and AB contributed to conception and design of the study. JM, RT, and DBo organized the database. DBo performed the statistical analysis and wrote the first draft of the manuscript. JM, TK, DBa, MB, CB, AB, VP, and TW wrote sections of the manuscript. All authors contributed to the article and approved the submitted version.

## Conflict of Interest

The authors declare that the research was conducted in the absence of any commercial or financial relationships that could be construed as a potential conflict of interest.
